# Genotypic analyses of IncHI2 plasmids from enteric bacteria

**DOI:** 10.1038/s41598-024-59870-2

**Published:** 2024-04-29

**Authors:** Suad Algarni, Dereje D. Gudeta, Jing Han, Rajesh Nayak, Steven L. Foley

**Affiliations:** 1 Division of Microbiology, National Center for Toxicological Research, Food and Drug Administration, 3900 NCTR Rd, Jefferson, AR 72079 USA; 2 Cell and Molecular Biology Program, University of Arkansas, Fayetteville, AR 72701 USA; 3 Office of Regulatory Compliance and Risk Management, National Center for Toxicological Research, Food and Drug Administration, 3900 NCTR Rd, Jefferson, AR 72079 USA

**Keywords:** IncHI2 plasmids, Antimicrobial resistance, Disinfectant resistance, Metal resistance, Mobile genetic elements, Enterobacteriaceae, Antimicrobial resistance, Bacterial genes

## Abstract

Incompatibility (Inc) HI2 plasmids are large (typically > 200 kb), transmissible plasmids that encode antimicrobial resistance (AMR), heavy metal resistance (HMR) and disinfectants/biocide resistance (DBR). To better understand the distribution and diversity of resistance-encoding genes among IncHI2 plasmids, computational approaches were used to evaluate resistance and transfer-associated genes among the plasmids. Complete IncHI2 plasmid (N = 667) sequences were extracted from GenBank and analyzed using AMRFinderPlus, IntegronFinder and Plasmid Transfer Factor database. The most common IncHI2-carrying genera included *Enterobacter* (N = 209), *Escherichia* (N = 208), and *Salmonella* (N = 204). Resistance genes distribution was diverse, with plasmids from *Escherichia* and *Salmonella* showing general similarity in comparison to *Enterobacter* and other taxa, which grouped together. Plasmids from *Enterobacter* and other taxa had a higher prevalence of multiple mercury resistance genes and arsenic resistance gene, *arsC*, compared to *Escherichia* and *Salmonella*. For sulfonamide resistance, *sul1* was more common among *Enterobacter* and other taxa, compared to *sul2* and *sul3* for *Escherichia* and *Salmonella*. Similar gene diversity trends were also observed for tetracyclines, quinolones, β-lactams, and colistin. Over 99% of plasmids carried at least 25 IncHI2-associated conjugal transfer genes. These findings highlight the diversity and dissemination potential for resistance across different enteric bacteria and value of computational-based approaches for the resistance-gene assessment.

## Introduction

Bacterial plasmids contain genes that impart selective advantage to the host species. Plasmids are key vehicles for the distribution of antimicrobial resistance (AMR), disinfectant and biocide resistance (DBR), and heavy metal resistance (HMR) elements among bacteria. A number of different resistance plasmid types have been identified in the *Enterobacteriaceae* that can be classified into various incompatibility (Inc) groups based on their predicted compatibility to coexist in a single bacterium^1^, with some of the key groups being IncA/C, FIB, HI1, HI2, I1, P and W, which are associated with resistance to multiple different antibiotics^2–4^. Among these, IncHI2 plasmids are an interesting group due to their large in size, typically greater than 200 kb, ability to encode for AMR, HMR and DBR, and transmission potential to facilitate resistance dissemination among enteric bacteria^5–9^.

The IncHI2 plasmids are typically associated with members of the family *Enterobacteriaceae*; including *Enterobacter, Escherichia, Salmonella, Klebsiella* and *Citrobacter* species^5,6,10–12^*.* Strains carrying these plasmids originate from a wide range of locations within Asia, Europe, North and South America, Africa and Australia^5,6,10,11,13–16^ and variety of sources, including human patients, foods, food animals, and the environment^5,17–20^.

These plasmids have gained significant public health interest due to their ability to carry AMR genes to a number of clinically important antimicrobials including colistin, carbapenems and other β-lactams, and quinolones^19,21–25^. Resistance to these antimicrobials has been on the rise globally, which has led to increased morbidity and mortality^26^. In the U.S., a study of hospitalized patients in 2017, found that 12.9% of *Enterobacteriaceae* were resistant to extended-spectrum β-lactamases (ESBL), 1.2% were resistant to carbapenems and 6.9% were multidrug resistant^27^. Plasmids were key contributors to the emergence of this resistance^26^.

IncHI2 plasmids often also carry HMR genes including for arsenic, copper, mercury, nickel, silver and tellurite^7,8,11,28^. The characterization of DBR and HMR is important since disinfectants, biocides and metal ions have been widely used for infection control and limiting microbial contamination in the environment^29–31^. Many IncHI2 plasmids are also known to be conjugative and can subsequently transmit the AMR, DBR and HMR genes from the resistant donor strains to previously susceptible recipients leading to the spread of a wide range of resistance elements in a single transfer event^9^.

With the wide availability of whole genome sequencing (WGS), the ability to assess the genetics of bacterial pathogens has been greatly enhanced though a wide variety of tools and bioinformatics approaches^32^. Computational programs such as PlasmidFinder, PubMLST, ResFinder, AMRFinderPlus, and IntegronFinder can identify and subtype plasmids and identify different genes associated with AMR phenotypes^33–36^. In addition, with the increasing access to long-read sequencing technologies, there is an enhanced ability to close plasmid sequences and determine the locations of resistance determinants and mobile genetic elements (MGEs), such as transposons, insertion sequences and integrons that make up the AMR mobilome that can facilitate the spread of genes among different plasmids and bacterial populations^37^. Understanding the genetic composition of plasmids is important to aid in epidemiological investigations and identifying measures to limit the spread of AMR.

The current project takes advantage of different bioinformatics tools available to assess the resistance genetics and molecular epidemiology of IncHI2 plasmid sequences available through the GenBank database to aid in the understanding the distribution of the plasmids among foods, the environment and humans. The IncHI2 plasmids identified were classified using plasmid multilocus sequencing typing (pMLST) and assessed to predict AMR, DBR, HMR and conjugal transfer gene content and the presence of integrons within the plasmids. The data allowed for evaluation of the distribution of resistance genes among the assembled plasmids from different bacteria taxa, and exploration of geographical and temporal sources. Understanding the epidemiology of the resistance mobilome can help with the development of strategies to limit the spread of multidrug and HMR carrying plasmids among bacterial pathogens in the environment, food animals and humans^37,38^.

## Methods and materials

### Selection of enteric plasmids for sequence analyses and data acquisition

The GenBank database was screened for IncHI2 plasmids based on their plasmid replicon sequence^39^. To select plasmid sequences for the project, the IncHI2-associated plasmid replicon target sequence described by Carattoli et al. (2005) was used for BLAST searching by narrowing the search to “Microbe” genomes and “Complete plasmids” in the Microbial Nucleotide BLAST database to return all sequences that were identified as highly similar sequences using default megablast settings^39,40^. These queries were conducted in December 2022 and returned 667 complete plasmid DNA sequences. Complete sequences for each plasmid were downloaded from GenBank in the FASTA and GenBank formats. These sequences were uploaded into the pMLST database program to categorize the plasmids into IncHI2 plasmid sequence types^33^. The identity, accession numbers, sequence type, bacterial host, and additional available metadata for each of the plasmids are provided in Table S1.

### Identification of resistance genes and integrons using GalaxyTrakr tools

The FASTA files for plasmids noted above were analyzed using AMRFinderPlus database V3.2.1^36^ with the AMRFinderPlus plugin within the GalaxyTrakr (https://account.galaxytrakr.org) operating environment as described previously^37^. AMRFinderPlus is a comprehensive database with 983 unique AMR genes, 82 unique HMR genes and 30 DBR genes. For several of these genes, there are multiple different variants of the genes in the database^36^. The output file describing the AMR, DBR and HMR genes was imported into Microsoft Excel and sorted by on sequence ID and gene type. For integron characterization, the analyses were performed using the IntegronFinder within GalaxyTrakr and the associated summary and integron annotation files were downloaded and imported into Excel for further analyses. The IntegronFinder output provides the number and locations of complete integrons and partial integrons containing either the insertion sites without the integrase nearby (CALIN) or containing the integrase without the insertion locations nearby (In0)^41^.

### Plasmid transfer gene analysis

To predict which putative plasmid transfer genes are present in the IncHI2 plasmids, the FASTA-formatted plasmid sequence files were submitted to the FDA Virulence and Plasmid Transfer Factor (VPTF) Database and searched with the Plasmid Transfer Factor Profile Comparison tool (https://virulence.fda.gov/tools/virulenceassess)^42^. The VPTF database contains sequences for type 4 secretion system genes for multiple incompatibility groups of resistance plasmids associated with enteric bacteria. The resulting output was exported to Microsoft Excel and included a detailed table containing the plasmid transfer genes, their percent identity to the reference sequence, number of mismatched base pairs, location of the gene in both the query and reference sequences, e-value, and bit score of the gene. Using the PivotTable function, the data was transformed to a presence/absence matrix with the e-value cutoff for inclusion in the table as 10^–3^.

### Statistical and phylogenetic analyses

To characterize the overall plasmid sequences, the GenBank formatted files for all 667 plasmids were uploaded into GalaxyTrakr and converted to GFF3 format required for analysis using Roary to determine the IncHI2 “plasmid pangenome”^43^. Default parameters were used for the analysis, with the exception of using a 90% presence cutoff for being considered part of the core set of genes. The Roary output was then used as an input into Scoary to facilitate comparison of phenotypic features to the genes present in the pangenome^44^. Features assessed included bacterial hosts, geographical location and sample type (human infection, animal, food, or environmental). Descriptive statistics including means and standard deviations were also calculated to facilitate comparison of the proportion of genetic elements among the plasmids collected from different hosts, locations, sample types and year of collection. For comparison of the distribution of AMR, DBR, and HMR genes across the different metadata variables, student *t*-tests and chi-square (χ^2^) test of independence was calculated in Microsoft Excel were used with statistical significance observed at *p* < 0.05. For these χ^2^ tests, data from *Klebsiella* plasmids were combined with the “other” taxa due to their relatively low numbers and the differences compared by drug class. For phylogenetic analyses, the presence/absence binary data was imported into the BioNumerics (Applied Maths, Austin, TX) program to assess the comparisons of the different profiles and sample characteristics. In BioNumerics, separate datasets were created for AMR, HMR and transfer-associated genes, while the DBR and integron-associated genes were combined due to genetic linkages and the limited data size (eight variables). For each group, the sample diversities were calculated using the Anderberg similarity coefficient^45^ and a dendrogram generated using the unweighted pair group means with averages (UPGMA) clustering techniques. In addition, the data were analyzed with BioNumerics to conduct principal component analyses (PCA) and generate minimal spanning trees based on the AMR, HMR and/or DBR/integron binary data. For the spanning tree analyses, the samples were identified by either bacterial genus, country of origin, year of isolation or sample type. A randomly selected plasmid representing each of the pMLST sequence types was selected for alignment using the BLAST Ring Image Generator (BRIG) program^46^. The nucleotide sequence for the ST1 representative (accession # NZ_LC590026.1) served as the reference sequence for the BRIG comparison and annotations.

## Results and discussion

**Figure 1:**
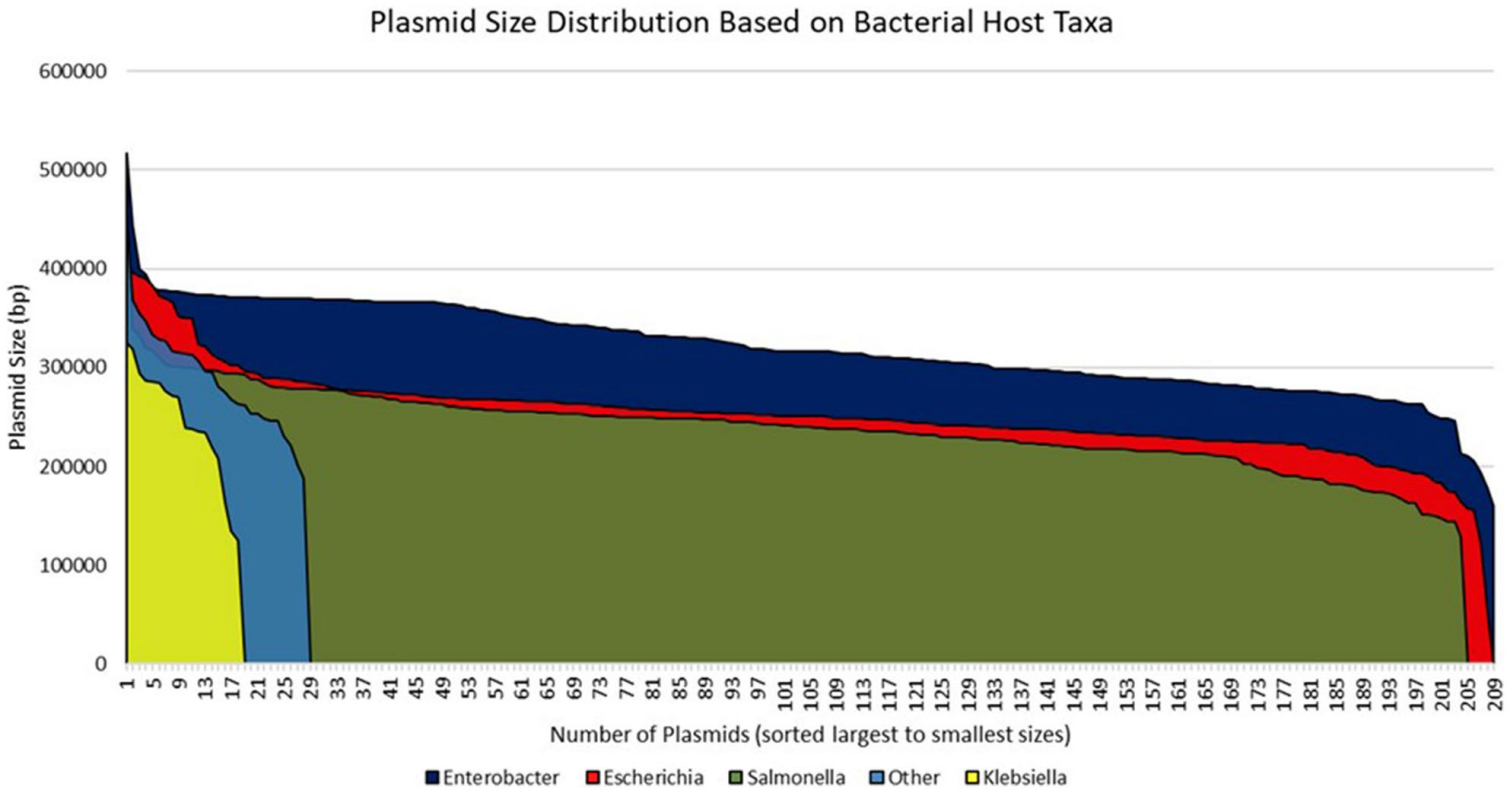
Reported sizes of the different IncHI2 plasmids across the different bacterial taxa. The plasmids are sorted left to right from largest to smallest for the particular taxon.

There were 667 IncHI2 plasmids that were detected using the study search parameters as of December of 2022. The details of the strains are provided in Table S1. Briefly, the dominant genera of the bacteria carrying the plasmids included *Enterobacter* (N = 209)*, Escherichia* (N = 208)*, Salmonella* (N = 204), and *Klebsiella* (N = 18), with the remaining taxa including *Citrobacter, Kluyvera, Leclercia, Raoultella, Serratia* and *Yersinia* species*.* On average, the sizes of the plasmids from *Enterobacter* were largest in size, averaging 319.5 kb (S.D. 45.6 kb), followed by the “other” taxa (289.5 kb; S.D. 56.3 kb), *Escherichia* (252.4 kb; S.D. 43.9 kb)*, Klebsiella* (244.9 kb; S.D. 57.8 kb) and *Salmonella* (238.9 kb; S.D. 41.0 kb) (Fig. 1). The predominant geographical source locations included China (N = 346), Japan (N = 76), United States (N = 39), Taiwan (N = 27) and Australia (N = 23). In several cases (N = 105), the year that the bacteria were collected was not provided; however, in those provided, the top years were 2018 (N = 88), 2016 (N = 78), 2017 (N = 68), 2019 (N = 63) and 2015 (N = 54). Isolates were collected from humans (N = 360), animals (N = 167), foods (N = 39), environment (N = 34), and unknown sources (N = 68). Strains that carried the IncHI2 plasmids were members of the *Enterobacteriaceae*; although *Serratia* and *Yersinia* have recently been placed in the new family *Yersiniaceae*^47^*.*

Most of the isolates originated from China (346/667, 51.9%) and the majority were from human patients (360/667, 54.0%). When reported, there was a fairly even distribution of strains isolated each year from 2015 to 2019 (range of 54–88/year). Additionally, there were circumstances where isolates from a certain country were predominated by a particular bacterial species. For example, of the 76 isolates from Japan, 56 (73.7%) were identified as *Enterobacter hormaechei.* Conversely, the *E. hormaechei* were isolated from a wide range of countries and across several years, so it may serve as a key taxon to look at IncHI2 resistance genetics in detail (discussed below).

**Figure 2:**
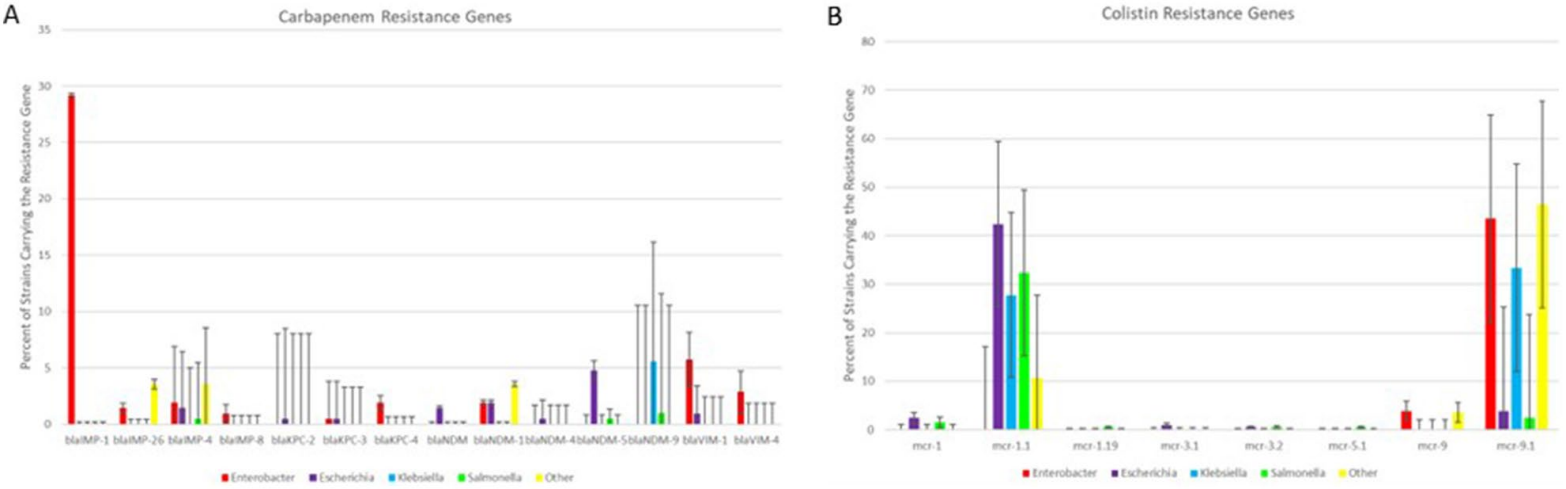
Percentage of isolates from a particular genus that has different AMR genes encoding resistance to different antimicrobial families: carbapenems (panel **A**) and colistin (panel **B**). The error bars indicate the standard deviation among the different taxa for the individual AMR gene displayed. Results for the other antimicrobial classes are shown in Figure S1.

The results of the AMRFinderPlus analysis identified 170 unique AMR genes in one or more of the 667 IncHI2 plasmid sequences analyzed, with the most common genes detect being *sul1* and *sul2* (sulfonamide resistance)*, aadA2, aph(6)-Id,* and *aadA1* (aminoglycoside/streptomycin)*, floR* (chloramphenicol)*,* and *tet(A)* (tetracycline); however, the distribution of genes varied across the different host bacterial taxa (Table 1 and Tables S2 and S3). For example, among the sulfonamide resistance genes there was a significant difference in distribution (χ^2^, *p* < 0.001), *sul1* was detected in plasmids isolated from the *Enterobacter* (77.0% positive)*, Klebsiella* (77.8%) and “other” genera (75.0%) compared to *Escherichia* (55.7%) and *Salmonella* (64.2%). Conversely, *sul2* and *sul3* were more commonly detected among those from *Escherichia* (40.9% and 50.5%, respectively) and *Salmonella* (60.7% and 49.0%) isolates compared to *Enterobacter* (20.6% and 1.4%)*, Klebsiella* (22.2% and 33.3%) and “other” genera (28.6% and 7.1%) (Table 1 and Figure S1A). Similar differences were seen with carbapenem and colistin resistance. For carbapenem resistance genes, *bla*_IMP-1_ was identified in 29.2% of plasmids from *Enterobacter*, but not found in the other plasmids. Other carbapenem resistance genes, including *bla*_NDM-5_ and *bla*_NDM-9_ were more common among plasmids from *E. coli, Salmonella* and *Klebsiella,* but not detected in those from *Enterobacter* (Fig. 2A)*.* For colistin resistance there was also a significant difference in the distribution of resistance genes (χ^2^, *p* < 0.001), *mcr-1.1* was more commonly detected in *Escherichia* (42.3%), *Klebsiella* (27.8%) and *Salmonella* (32.4%) in comparison to not being detected among the *Enterobacter* and less commonly among the “other” genera (10.7%). In contrast *mcr-9.1* was detected in *Enterobacter* (43.5%)*, Klebsiella* (33.3%) and “other” genera (46.4%), while only in 3.8% and 2.4% of plasmids from *Escherichia* and *Salmonella*, respectively (Fig. 2B). Likewise, the gene selection for several of the other drug classes showed significant taxa level distribution differences as well (χ^2^, all noted differences *p* < 0.001), including tetracycline genes (e.g. *tet(A)* vs. *tet(B)*), quinolones (e.g. *oqxA* and *oqxB* genes vs. *qnrA* and *qnrB* genes in *Escherichia* and *Salmonella* vs. *Enterobacter*), trimethoprim (e.g. *dfrA12* vs. *dfrA19*), macrolides (e.g. *ere(A)* vs. *mph(A)* in *Enterobacter* vs. *Escherichia* and *Salmonella*), phenicols (e.g. *catA* vs. *floR*), and β-lactams (e.g. *blaOXA-1* vs. *blaSHV-12*) (Figures S1B-G).

**Table 1:**
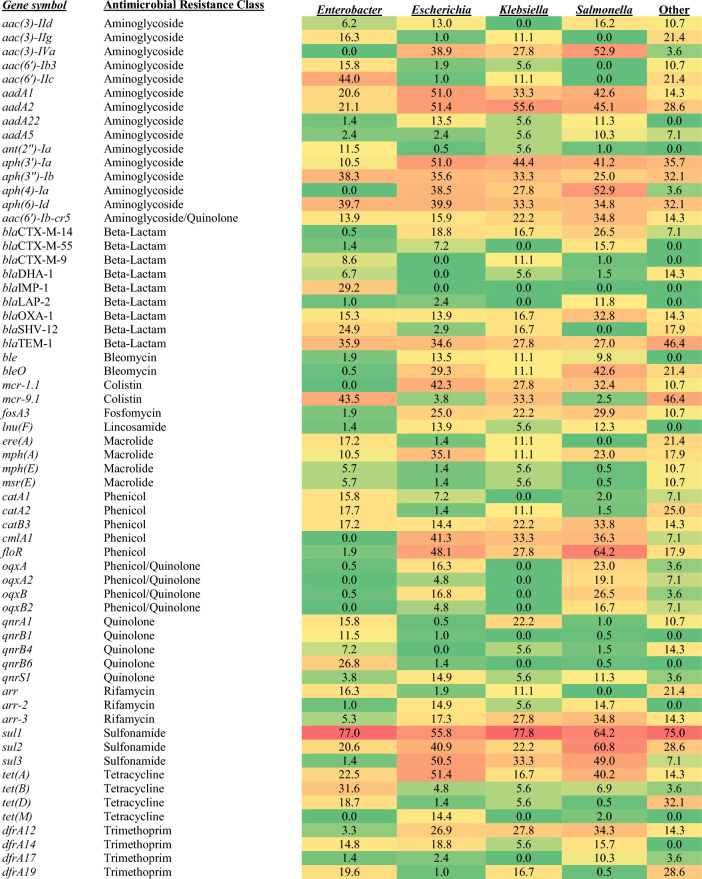
Proportion of plasmids from the respective Genus that carried specific AMR genes.

Color scheme is based on the proportion of plasmids that carried the AMR gene ranging from green (lowest) to yellow to red (highest). Genes shown are in at least 10% of one of the taxa. The complete data are in Table S2.

The characterization and reporting of the resistance genes have been the focus of many manuscripts describing IncHI2 plasmids. A search of PubMed on “IncHI2 plasmids” identifies hundreds of papers, many of which have focused on the carriage of colistin, carbapenems and other β-lactam (primarily ESBL) resistance genes due to their importance for clinical medicine^19,21–25,48,49^. For example, Aoki et al. (2018) characterized *bla*_IMP-1_-carrying *Enterobacter* species isolated from patients in Japan and found that this carbapenem resistance gene was often carried on pST1 IncHI2 plasmids. These findings correspond to the results of the current study that found that the majority (59/61, 96.7%) of the *bla*_IMP-1_-carrying plasmids were isolated from *Enterobacter* in Japan^50^. Similarly, Timmermans et al. (2021) examined colistin resistance in *E. coli* isolated from different food animals in Belgium during the years of 2012–2016. Of the colistin resistant isolates in their study and the current study, *mcr-1.1* was detected on IncHI2 plasmids in *E. coli* across multiple years^51^. These papers represent a small fraction of the IncHI2 literature focused on the characterization of these important resistance genes from the plasmids in bacteria from varied geographical locations, host sources and time periods, highlighting the global spread of IncHI2 plasmids^23,52–54^.

A potentially concerning reality is that in many cases IncHI2 plasmids are co-located with other resistance plasmids that carry overlapping genes encoding the resistance elements detected on the IncHI2 plasmids in the study. Khine et al. (2023) showed that *mcr-1.1* in *E. coli* can be carried on IncI2 plasmids flanked by different insertion sequences. These plasmids could potentially facilitate transfer from the IncI2 plasmid to the IncHI2 plasmid if they are co-located in the same bacterium. The IncHI2 plasmids have several regions with insertion sequences that can lead to variability in the gene content of the different plasmids^12,55^. The most conserved regions in the IncHI2 plasmids appears to be the transfer regions of the plasmid. The presence of the transfer regions provides that many of these plasmids are conjugative and can lead to the spread between different bacteria in the same niche^56^.

**Figure 3:**
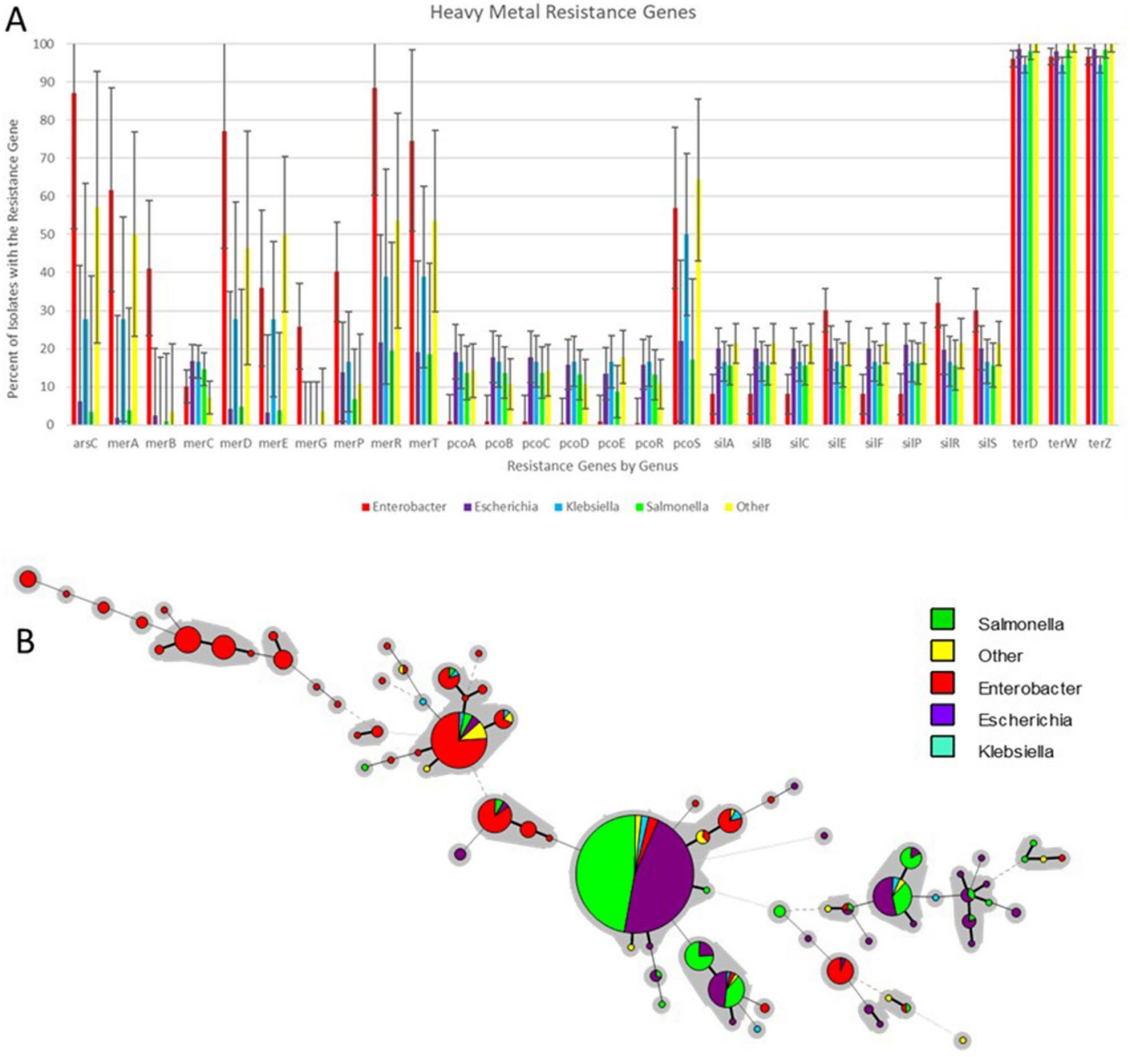
The percentage of isolates from a particular genus that have different HMR genes displayed (panel **A**). The error bars indicate the standard deviation among the different taxa for the individual AMR gene displayed. The lower panel (panel **B**) provides the results of minimum spanning tree analyses based on all the HMR genes identified among the plasmids in the study. The size and proportions in the circles are representative of the number of isolates within the group with identical gene profiles. The grey shading around the circles represents partitioning groups based on relative similarity of the groups.

In addition to the AMR genes, AMRFinderPlus was used to identify the HMR and DBR genes in the IncHI2 plasmids. There were 36 HMR genes detected, including multigene operons for arsenite efflux (*ars*), copper resistance (*pco*), copper/silver resistance (*sil*), mercury resistance (*mer*), nickel resistance (*ncr*), and tellurite resistance (*ter*) (Fig. 3). The most commonly detected resistance genes were the tellurite resistance genes *terD, terW* and *terZ*, which were detected in at least 94% of the strains from each genus. The arsenic resistance gene *arsC* was present in 87.1% of the IncHI2 plasmids isolated from *Enterobacter*, from 57.1% of the “other” taxa and 27.8% of the *Klebsiella. arsC* was less commonly detected in the plasmids from *Escherichia* (6.3%) and *Salmonella* (3.4%). There was a significant difference in distribution of many of the mercury resistance genes across the taxa, with many of the genes more common in the plasmids from *Enterobacter*, the “other” taxa and *Klebsiella* in comparison to those from *Escherichia* and *Salmonella* (Fig. 3A)*.* Similarly, the distribution differences among the copper resistance genes (*pco* operon) were significant (χ^2^, *p* < 0.001), as each gene was less commonly detected in *Enterobacter* plasmids, with the exception of *pcoS,* where the gene was more frequently detected. Minimum spanning tree analyses indicated distinct lineages of *Escherichia* and *Salmonella* clustered together (Fig. 3B). Among the DBR genes there was also significant difference in the distribution of genes (χ^2^, *p* < 0.001), *qacEΔ1* was more commonly detected in *Enterobacter* derived plasmids (75.6%) than those from *Escherichia* (39.9%) and *Salmonella* (44.1%; Figure S2). This higher proportion of *qacEΔ1* in *Enterobacter* may correspond with the higher proportion of *sul1* genes in the taxa, as both are common components of many of the class 1 integrons^57^. In contrast, *qacL* was present at much lower levels in *Enterobacter* plasmids (0.5%) compared to those from *Escherichia* (44.2%; Figure S2)*.*

**Figure 4:**
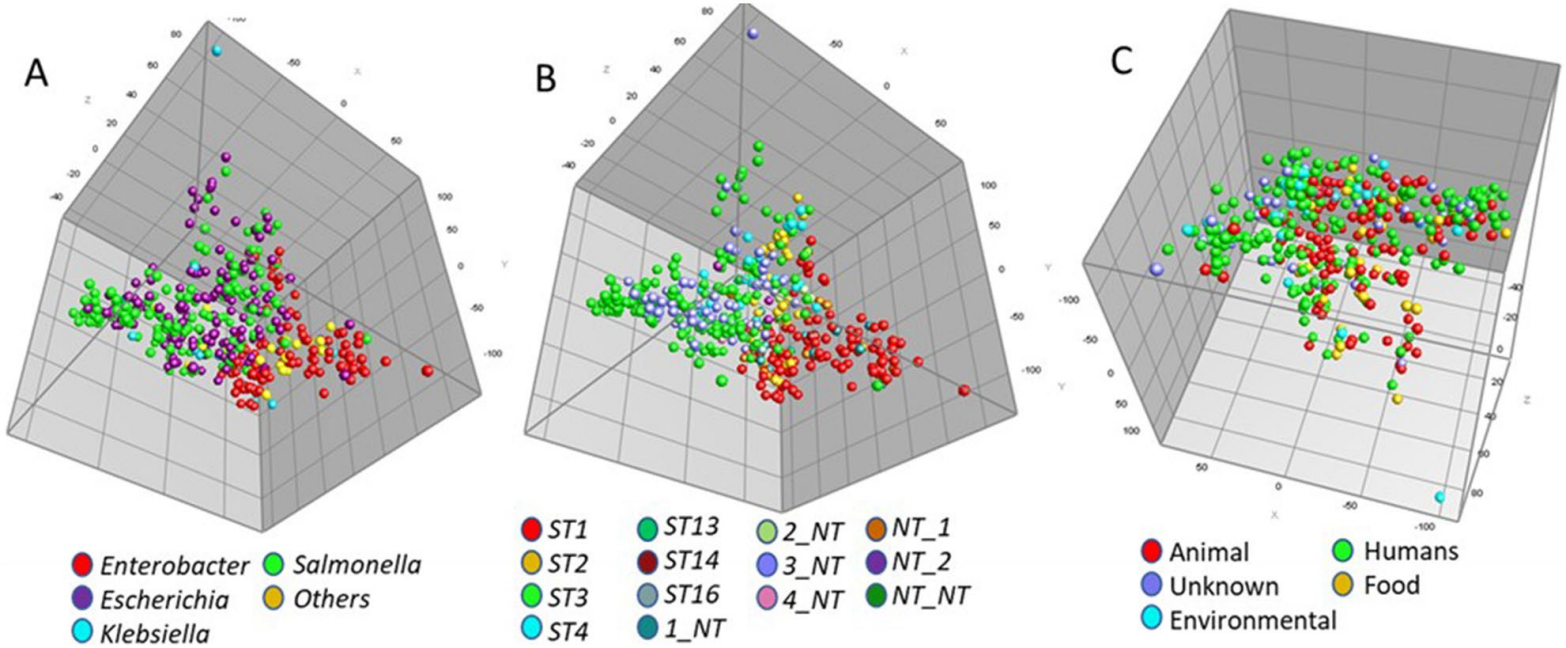
Principal components analysis (PCA) plots of AMR gene presence/absence data for the 667 different plasmid sequences. The plasmids were characterized by: bacterial host taxa (panel **A**); plasmid sequence type (panel **B**); and sample type (panel **C**). Each of the circles represents a distinct genotype and may contain multiple members from the identified groups. The PCA analyses are displayed in 3D plots rotated to maximumly visualize the diversity of the groups.

Further analyses of the AMR, DBR and HMR resistance genes in relation to plasmid sequence type (pST), host bacterial genera/species, geographical and temporal origin were assessed using different comparative approaches (Fig. 4). When assessing the pST results the predominant STs were ST3 (N = 217), ST1 (N = 210), ST2 (N = 59) and ST4 (N = 33) (Table 2 and Table S1). The pMLST typing is based off two different loci (smr0018 and smr0199) and there were 135 plasmids that lacked one of the two loci, with the largest group having the allele 3 for smr0018 and no allele for smr0199 (N = 84). ST3 was made up of the smr0018 allele 3 and smr0199 allele 2; thus these 84 incomplete ST (3_NT) shared the smr0018 allele sequence with ST3^58^. ST3 and 3_NT were most commonly identified in plasmid from *Salmonella, Escherichia* and *Klebsiella* (Table 2); whereas ST1 and other STs with smr0018 allele 1 (1_NT, ST14, and ST16) where most commonly detected from *Enterobacter,* other taxa, and *Klebsiella* plasmids. These plasmids were the largest sized IncHI2 plasmids, each group averaging over 280 kb in size, while all other STs (with the exception of NT_1) averaging less than 276 kb in size (Figure S3). ST2 was spread across each of the different genera, except for the other taxa plasmids. Of the *Enterobacter* strains that were ST2, the majority (8/11, 73%) were species other than *E. hormaechei*, which was the most common species among the *Enterobacter* found in the study*.*

**Table 2:**
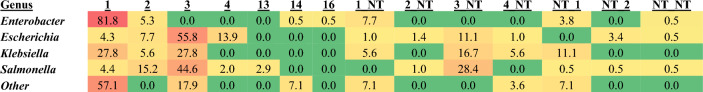
Proportion of IncHI2 plasmid multilocus sequence typing (pMLST) types detected among the plasmids from their respective Genera.

Color scheme is based on the proportion of plasmids within a specific pMLST type in the different bacterial taxa ranging from green (lowest) to yellow to red (highest). Those identified as "NT" did not contain either the smr0018 allele and/or smr0199 allele, respective.

**Figure 5:**
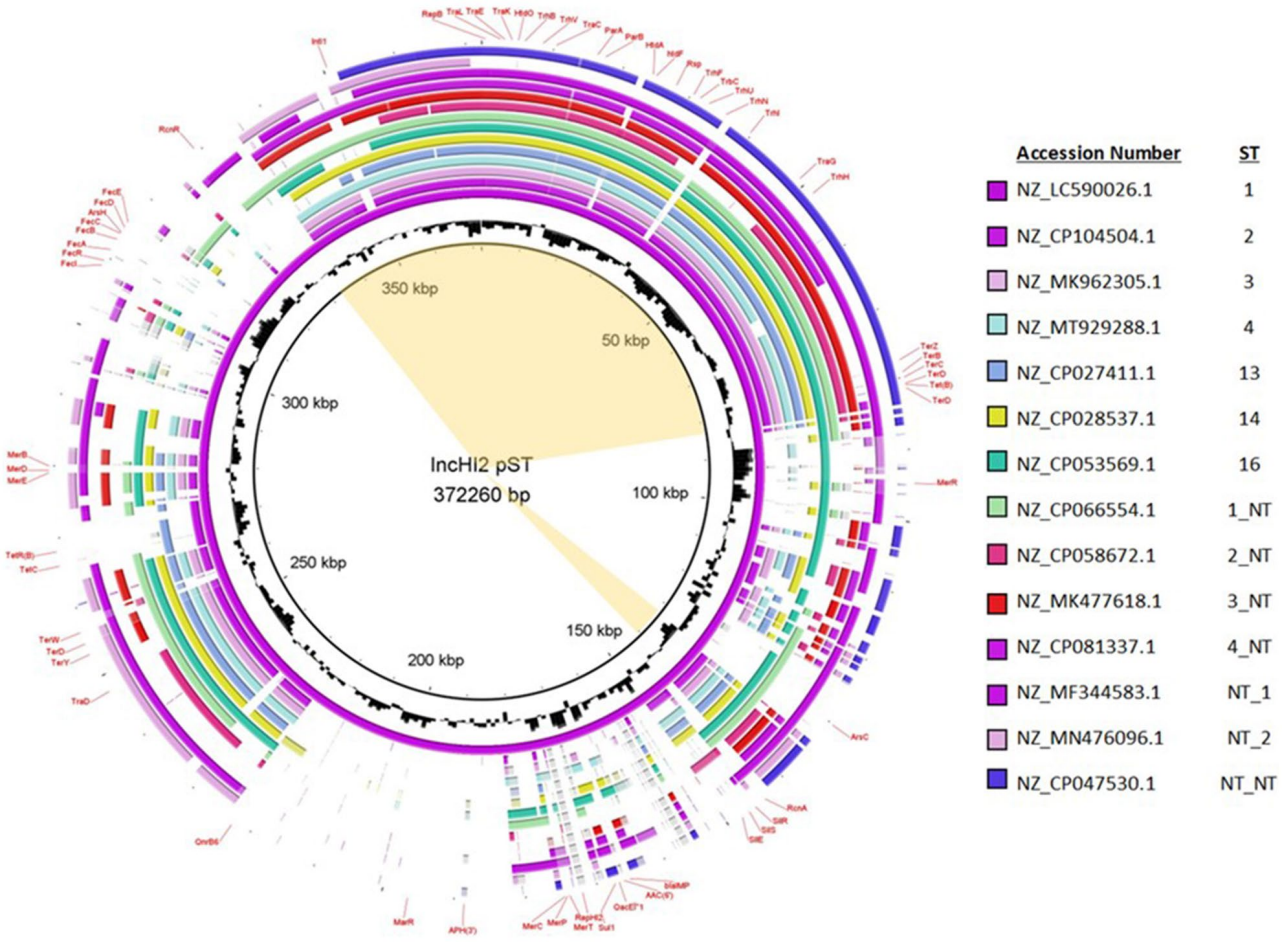
Alignment of randomly selected plasmids representing each of the pMLST sequence types identified using the BRIG program. The inner ring is the GC content of the reference plasmid, which was the ST1 representative (accession # NZ_LC590026.1). The inner purple ring is the representative reference sequence for the BRIG comparison and annotations. The light yellow regions of shading in the center of the plasmid circles represents the core plasmid genetic regions, with these genes being present in at least 90% of the IncHI2 plasmids in the study as determined by Roary analysis.

The BRIG alignment of representatives of each the STs indicated highest levels of conservation appeared to be among the transfer region genes and other genes associated with the plasmid backbone (Fig. 5). When looking at the plasmid pangenome prediction based on Roary analysis, there were 70 genes that were present in 90% of the plasmids, which could be viewed as the “core” sequences of the IncHI2 plasmids. This 90% threshold, which is somewhat more lenient than for bacterial genomes, was chosen due to the high level of sequence plasticity among plasmids. The genes that made up this core genome included many of the transfer-associated genes, plasmid toxin/antitoxin genes and the tellurite resistance operon which correspond to the regions spanning for about 335 kbp to 85 kbp and 132 kpb to 145 kpb in the BRIG diagram in Fig. 5. The accessory genome, where a gene was detected in at least 2 plasmids, but outside the core genes, included 2,644 genes. Many of these accessory genes appear to related to plasmid recombination (e.g. transfer genes from other plasmid types) or are variants of the same gene, which is possibly due to the default 95% amino acid similarity cutoff used to define separate genes in Roary^43^. The accessory genome includes most of the resistance genes observed among the plasmids, which is consistent with the diverse results across the different taxa. The Scoary analyses also showed demonstrated differences in the gene content of the plasmids from the different bacterial taxa (Supplemental Data File). This phenomenon was most evident with the plasmids from the *Enterobacter* in which there were multiple genes associated with membrane transport and gene regulation that were only found in the IncHI2 plasmids from *Enterobacter*. As further data on geographic and source locations were included in the puzzle, it was shown that these more than 30 genes were associated (Scoary, *p* < 0.001) with a group of plasmids isolated from *E. hormaechei* isolated from human patients Japan across multiple years (Supplemental Data File).

Among the HMR genes, several of the *mer* and *ter* genes were conserved across many of the analyzed STs, which is not surprising since these are among the most commonly detected HMR genes among all the plasmids (e.g., *terD, terW* and *terZ* were detected in about 95% of the plasmids in the study; Fig. 3). Interestingly, when the sample types were examined, the plasmids from strains collected from environmental sources had on average a higher number (N = 10.3) of HMR and DBR genes than those from human patients (N = 9.3), foods (N = 9.1) and animals (N = 8.3). While these differences are not statistically significant, they do provide evidence that environmental exposures to heavy metals and disinfectants may provide selective pressure for resistance development.

When examining the distribution of AMR genes across the different taxa using PCA analyses, the aforementioned grouping of taxa is evident (Fig. 4A), with the *Escherichia* and *Salmonella* largely separating from the three other taxa. These grouping differences also pertain to the different plasmid STs (Fig. 4B), which is not too surprising since specific STs were typically associated with the different taxa (Table 2). There appears to be overlap of the AMR profiles is with the different sample types, with plasmids originating from human patients extensively overlapping with those originating from food, animals and the environment (Fig. 4C). When looking at the specific taxa, among the *Salmonella* strains, there was separation of the plasmid AMR gene profiles based on the pST. The majority of the plasmids fell into ST3 or the related 3_NT (148/204, 72.5%). Of the plasmids from other STs, each tended to cluster together with the other plasmids of the same ST. All six ST13 plasmids were isolated in the U.S. and had identical AMR gene profiles, yet they were isolated in different years (2002–2012) and different sources (animal and human; Figure S4). A majority of the ST2 plasmids (24/31, 77.4%) formed a separate cluster from most of the ST3 plasmids, while four of the remaining ST2 plasmids clustered with the ST4 and ST13 plasmids and two other ST2 plasmids shared a distinct gene profile with an NT_2.

Among the major genera carrying the IncHI2 plasmids, plasmids from *Escherichia* appear to be the most diverse, both in terms of AMR gene profiles (Fig. 4B) and pSTs. Plasmids from both *Salmonella* and *Enterobacter* had multiple groupings of more than five plasmids sharing common AMR gene profiles (Figures S4 and S5), whereas for *Escherichia* the largest group sharing a common AMR profile contained only five plasmids (Figure S6). Like was observed with the plasmids from *Salmonella,* there was some clustering of ST2 and ST4 for *Escherichia* plasmids, although the clusters were not as tight, which may be due to the *Escherichia* plasmids carrying larger numbers of different resistance genes than the *Salmonella* plasmids (118 vs. 91; Figure S6)*.*

When we look at the host bacteria at the species level, all of *Salmonella* are *S. enterica* and a majority of the *Escherichia* are *E. coli* (197/208, 94.7%); whereas for the 209 *Enterobacter* strains*,* there is more diversity with 148 (70.8%) being *E. hormaechei*, 24 (16.3%) *E. cloacae,* 10 (4.8%) *E. asburiae* and remaining 17 (8.1%) spread across at least 6 other species. The *E. hormaechei*-associated plasmids are an interesting subset to explore due to their wide geographic and temporal distribution across a variety of host sources (Figure S5). When looking at the countries of origin of the strains carrying the plasmids, there are some clonal AMR clusters that were associated with Japan, The Netherlands, Canada, Mexico, and The Czech Republic. In several of the cases, plasmids with clonal AMR gene profiles were isolated across multiple years; for example, strains carrying plasmids from the Japanese cluster isolated from human patients from 2007 to 2020 (Figure S5).

**Figure 6:**
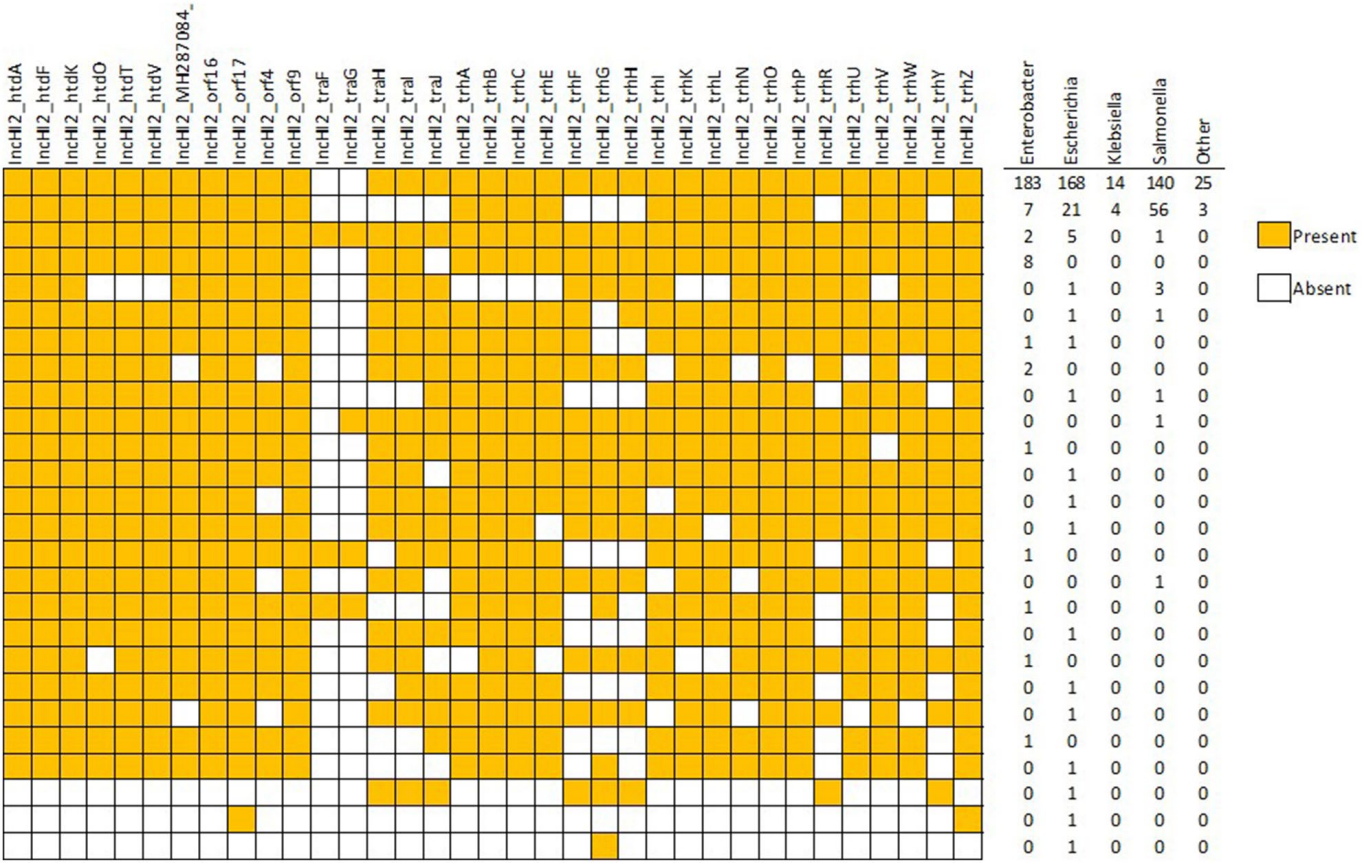
The presence and absence of the different IncHI2 transfer-related genes among the isolates in the study. The columns to the left of the figure are the numbers of plasmids originating from the different strains that carried the transfer gene profiles.

The conjugal transfer of IncHI plasmids is interesting in a number of facets, including that the optimal conjugation temperatures, at least in vitro, appears to be in the range of 25 to 30 °C, although the plasmids can transfer at lower frequency at 37 °C^57,59^. These lower optimal conjugal transfer temperatures could lead to environmental locations playing an important role in spread of these plasmids. Additionally, the co-transfer of IncHI2 plasmids with other plasmid types can impact the efficiency of the plasmid transfer, potentially indicating a potential co-regulation of transfer and creating opportunities of cointegration of multiple plasmid-associated genes^57^. This cointegration of genes across plasmid types may lead to increase plasmid diversity and acquisition of resistance elements. When assessing the IncHI2-associated transfer genes specifically, the predominant genotype included all the genes except for *traF* and *traG* (N = 530, 79.5%; Fig. 6). This genotype was identified in plasmids from across the globe representing all genera, pST, and sources. The second most common genotype included all the genes except *traF-traJ, trhF-trhH, trhR,* and *trhY* (N = 91, 13.6%). Each of these plasmids also lacked the smr0199 allele as part of the pMLST scheme, including 1_NT, 2_NT, 3_NT, and 4_NT, which is likely due to this allele being located to close to these transfer genes that are missing in this group of plasmids^60^. The host isolates for these plasmids came from multiple continents, across a range of years and from all source categories, but were predominantly from *Salmonella* and *Escherichia* isolates.

The impacts of the differences in the transfer genes were not assessed in the current project; however, it is curious what the impact on plasmid transfer would be for those plasmids lacking multiple genes. Zhang et al. (2022) found that the deletions of the transfer gene region, and some of the AMR genes, may be a compensatory mechanism that reduces the cost of maintaining the plasmids in the host strain. Their experiments showed that strains carrying the IncHI2 plasmids that had the regions deleted during serial passaging of the bacteria had reduced fitness costs compared to the strains that did not undergo the deletions within the plasmids^61^. Part of the factors that appeared to contribute to the evolution of the plasmids with the compensatory gene deletions was the exposure to different antimicrobial agents. The tradeoff of the reduction of genes for the increased fitness was the likely reduction in the ability for the horizontal gene transfer of the plasmids like those lacking the smr0199 locus mentioned above (Fig. 6). The increase fitness and decreased ability for horizontal plasmid transfer may contribute to some clonal plasmids persisting in a bacterial species in a common geographical region over several years.

Class 1 integrons were detected in 75% of the plasmids (N = 498) (Figure S2). Based on the IntegronFinder and AMRFinder analyses, the integrons carried diverse sets of resistance genes, with some of the integron elements appeared complex with multiple resistance cassettes and integration sequences (Table S4). Zhao et al. (2018) noted that the integrons among the IncHI2 plasmids can be quite diverse, even among plasmids within the same pST and host taxa. The presence of these integrons allows for the accumulation of AMR genes within these plasmids facilitating the multidrug resistance phenotype and potential spread of resistance^62^.

While the study shows the importance of IncHI2 plasmids to AMR, DBR and HMR development and spread, the study has some caveats to consider in the overall interpretation. The first is that the results are based on the complete plasmid sequences that were available in GenBank as of December of 2022 which are reliant on the types of sequences completed and uploaded to the database. The host bacteria tend to skew to those associated with human illness and those encoding resistance, as these plasmids may have a higher public health significance. Secondly, the geographic origin of the sequences tended to be most heavily dominated by a few countries that have more heavily invested in long-read DNA sequencing technologies that are important for closing of the plasmids or possibly sets of bacteria/plasmids associated with individual focused studies. These potential issues will likely diminish in the near future with the increasing availability of new sequencing technologies and subsequent availability of high-quality closed plasmids. Even with these potential limitations, the study provides a valuable resource for the assessment of the IncHI2 plasmids in enteric bacteria across a wide range of species, geographical locations and time points that is important to understand the epidemiology of IncHI2 plasmids. The IncHI2 plasmids warrant continued surveillance to determine whether shifts in resistance profiles and bacterial host ranges can lead to increased public health risks due to the potential spread of large numbers of different resistance genes that can be carried on these plasmids.

### Supplementary Information

Supplementary Information 1.

Supplementary Information 2.

Supplementary Information 3.

## Data Availability

All data utilized in this study are publicly available through NCBI GenBank. GenBank Accession Numbers are provided in Table S1.
