# Supplementary material for: Genotypic analyses of IncHI2 plasmids from enteric bacteria
Source: Sci Rep. 2024 Apr 29;14:9802. doi: 10.1038/s41598-024-59870-2 (PMC11058233; doi:10.1038/s41598-024-59870-2)
Supplement: Supplementary file 1 — Supplementary Information 1. [file 41598_2024_59870_MOESM1_ESM.docx]

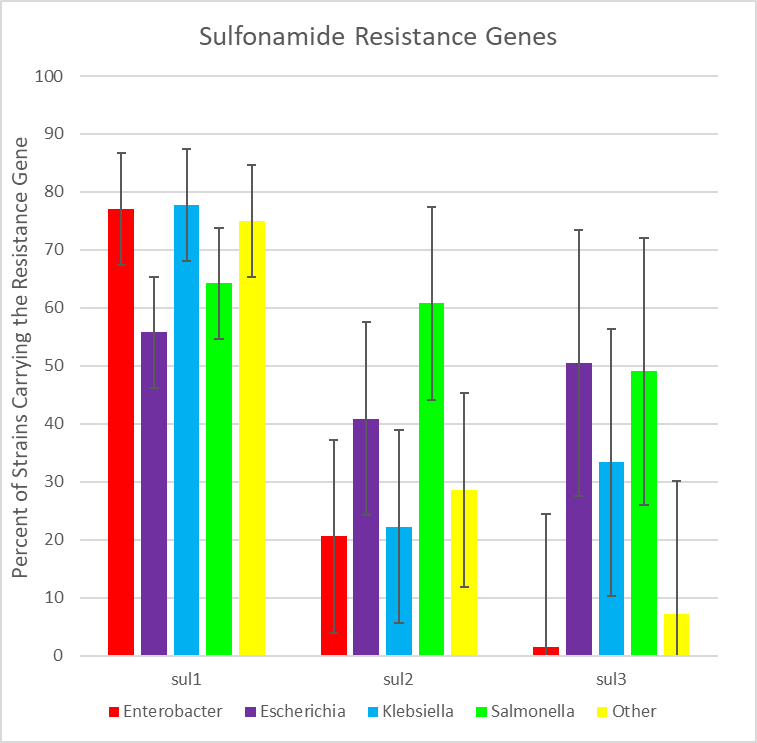
**
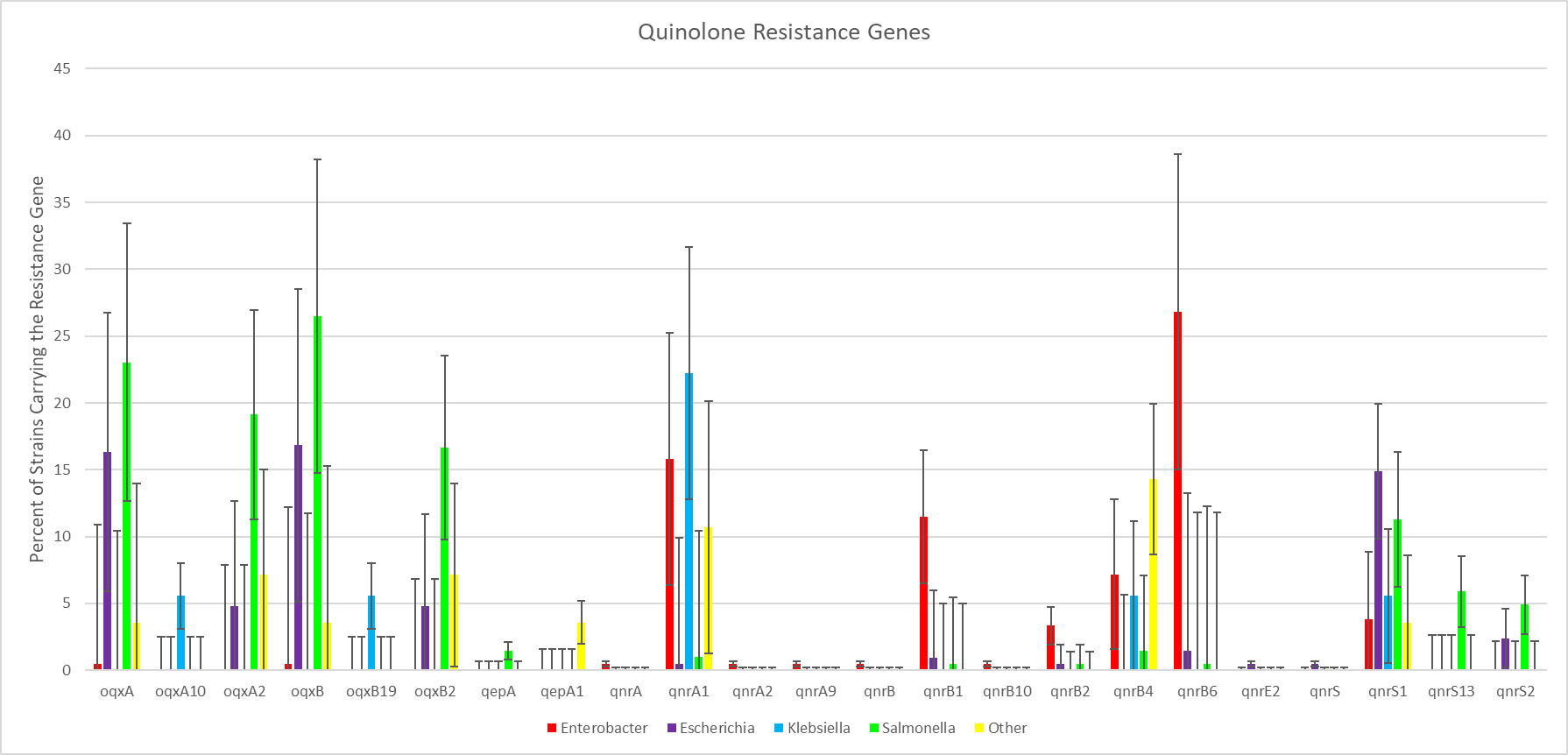
**

A


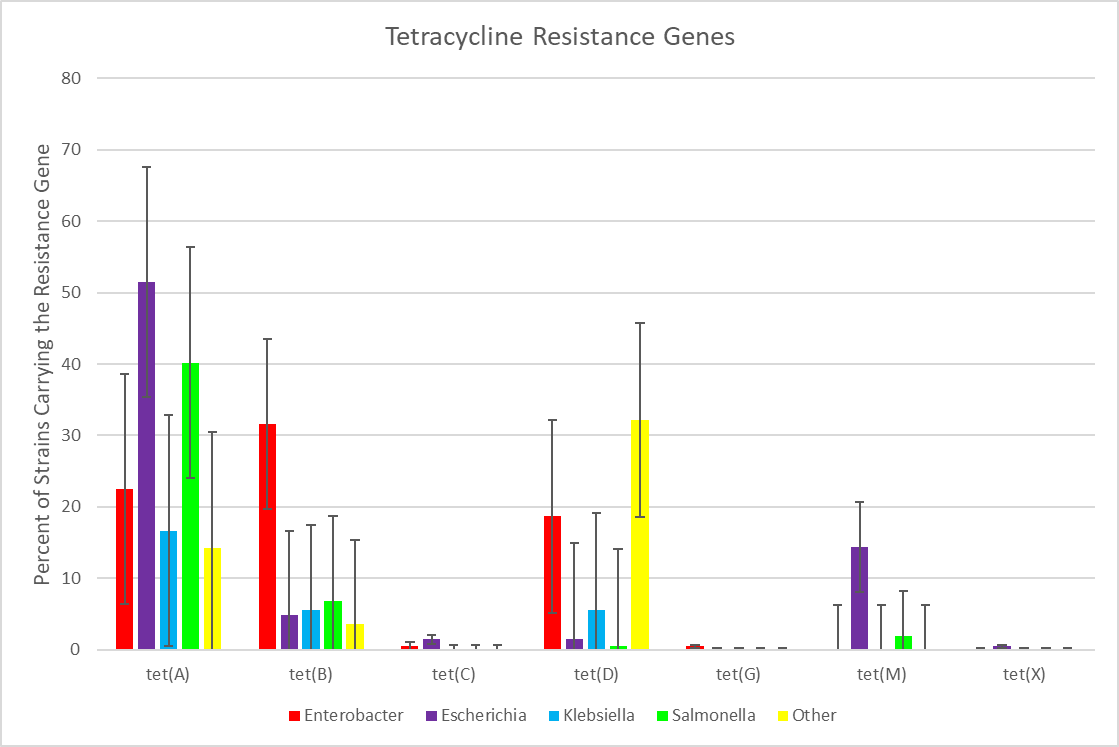


B

C

D

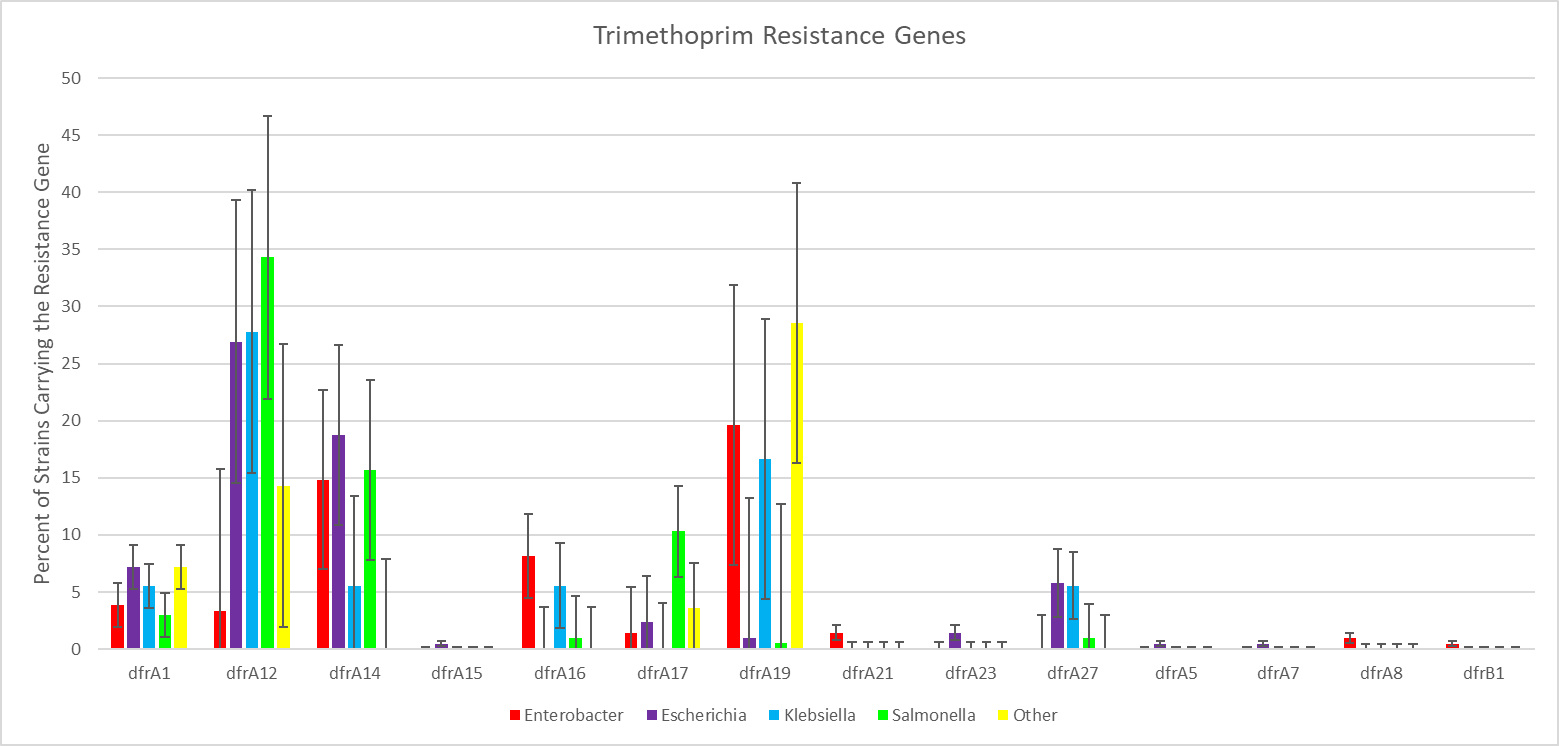


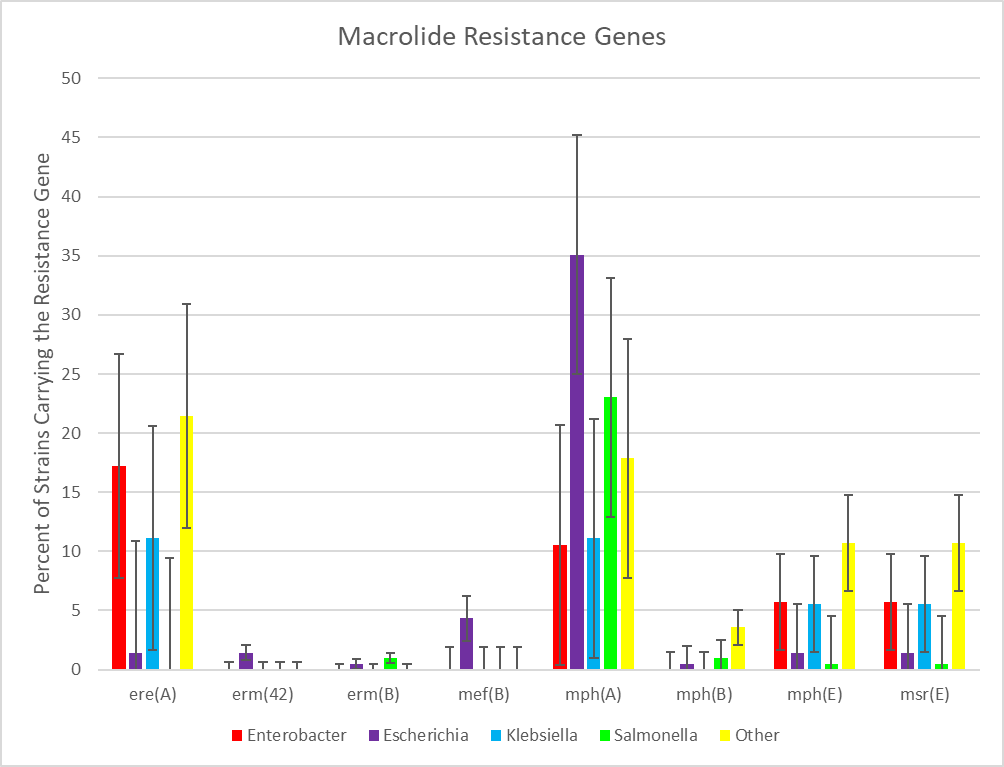

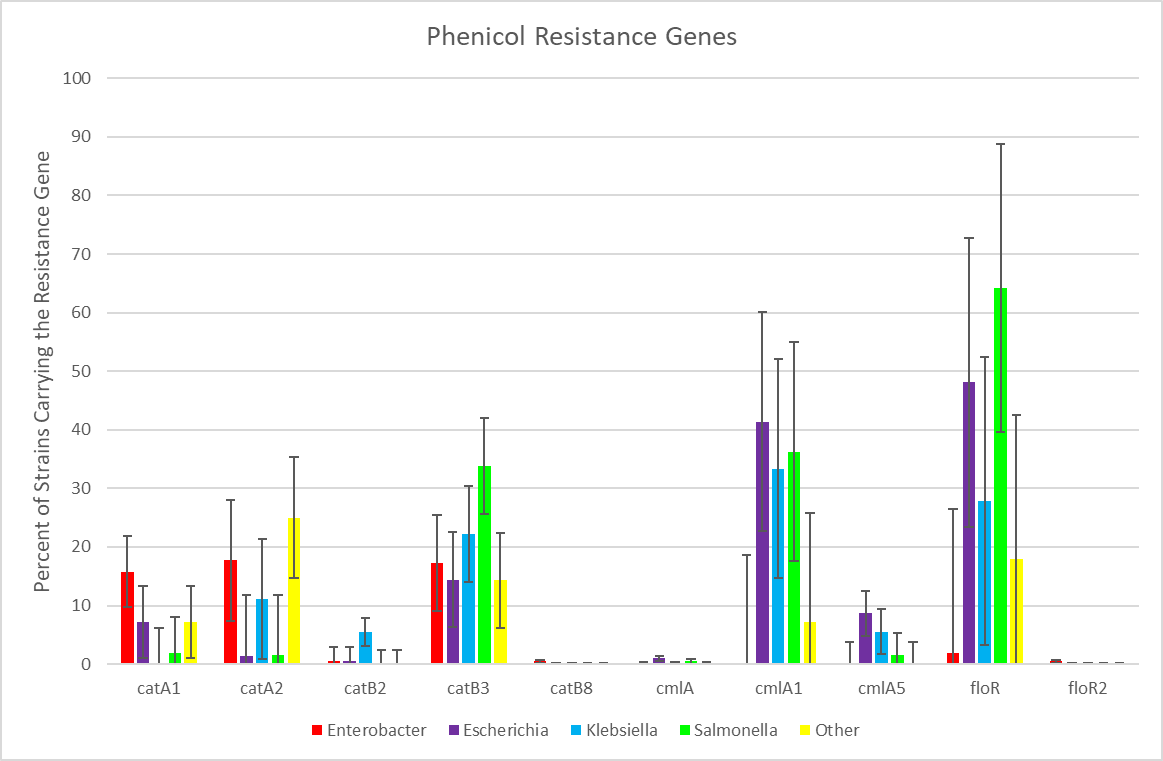


G

F

E

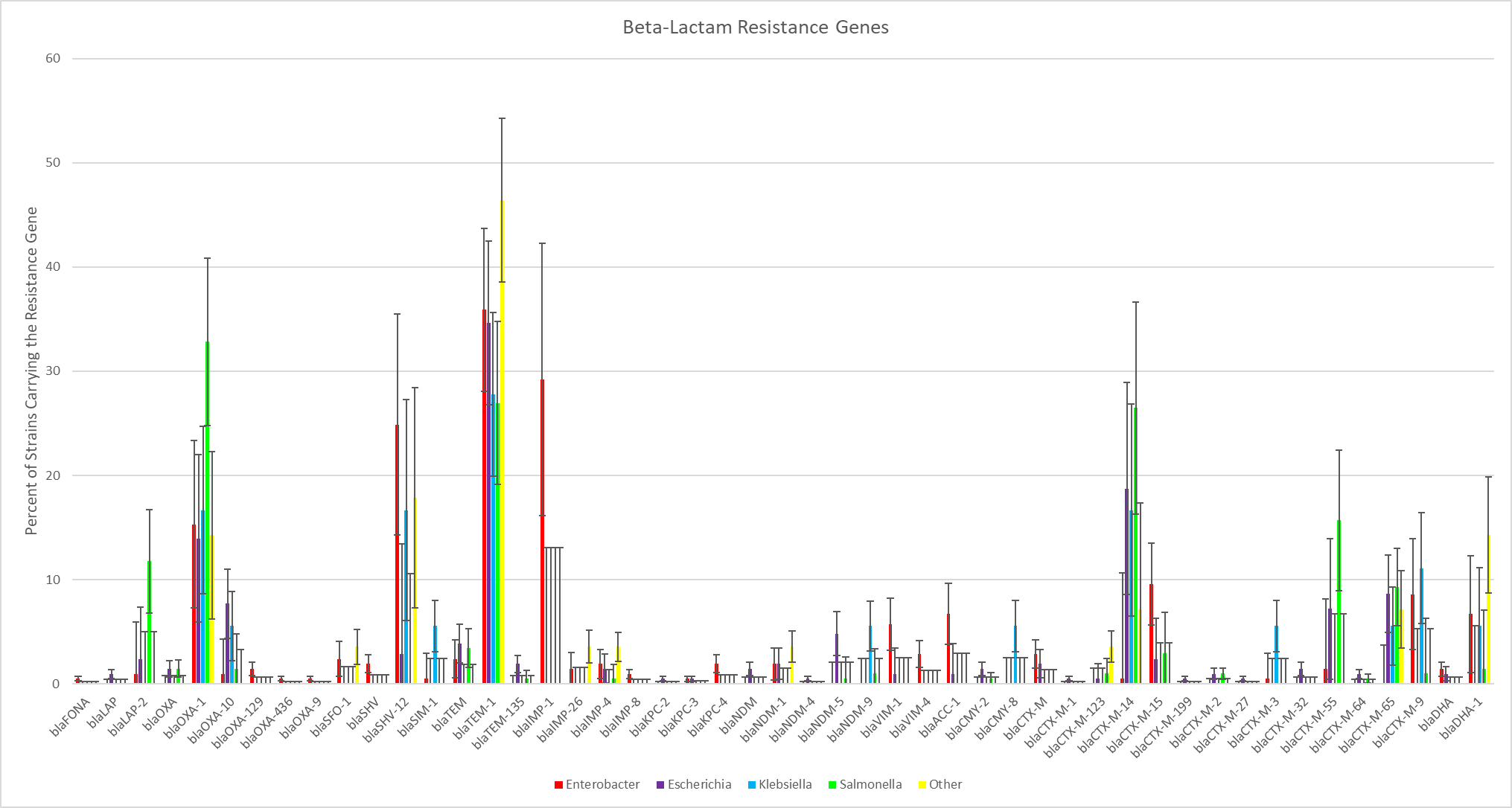


**Figure S1.** Percentage of isolates from a particular genus that has different AMR genes encoding resistance to different antimicrobial families: A. sulfonamides, B. tetracyclines; C. quinolones; D. trimethoprim; E. macrolides; F. phenicols; and G. β-lactams. The error bars indicate the standard deviation among the different taxa for the individual AMR gene displayed.


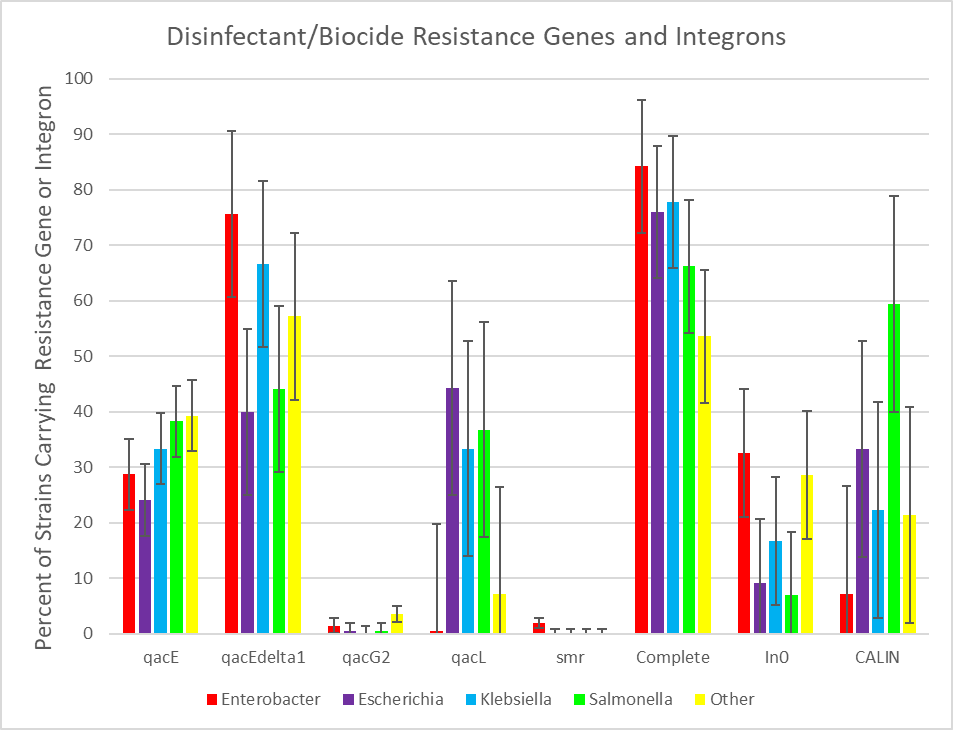


**Figure S2.** Percentage of isolates from a particular genus that has different DBR genes and integron related sequences. For the integron sequences, those labelled as “Complete” are identified complete integrons, carrying all of the essential elements of the integron; whereas, “In0” are those integron-related elements with only the integrase gene elements (lack the *attC* sites), and those identified as “CALIN” are those with the cluster of *attC* sites lacking integrase nearby elements.


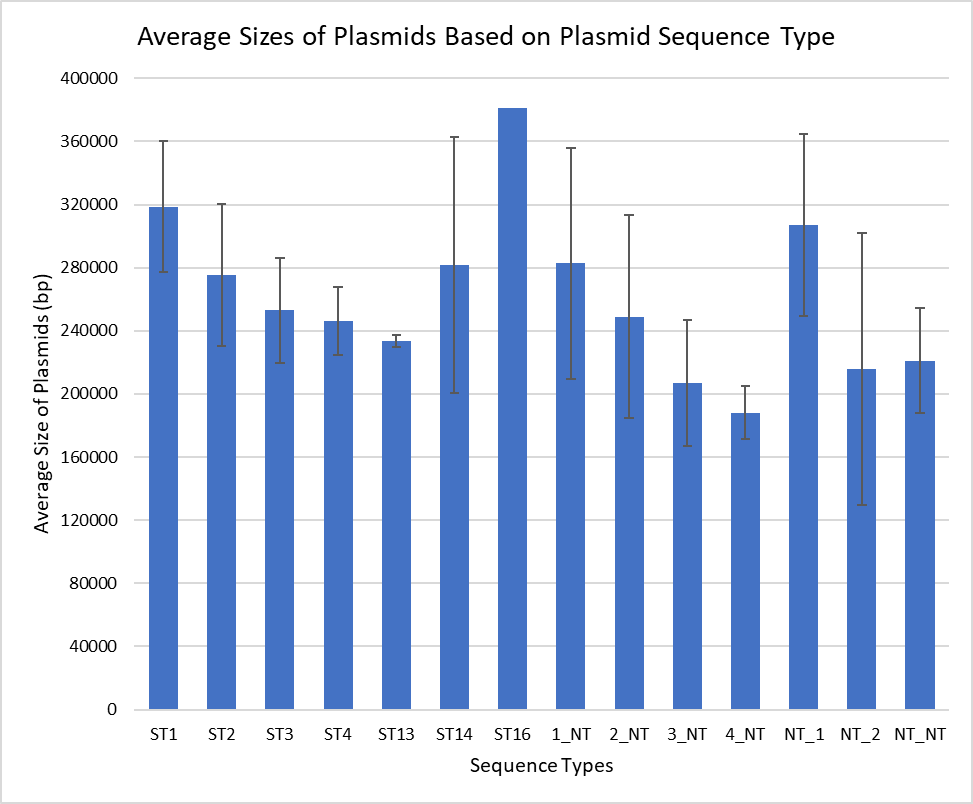


**Figure S3.** Average sizes of the plasmids based on their predicted plasmid sequencing types. The error bars represent the standard deviation of the data within the different groups. ST16 has only a single member, thus no standard deviation bars.


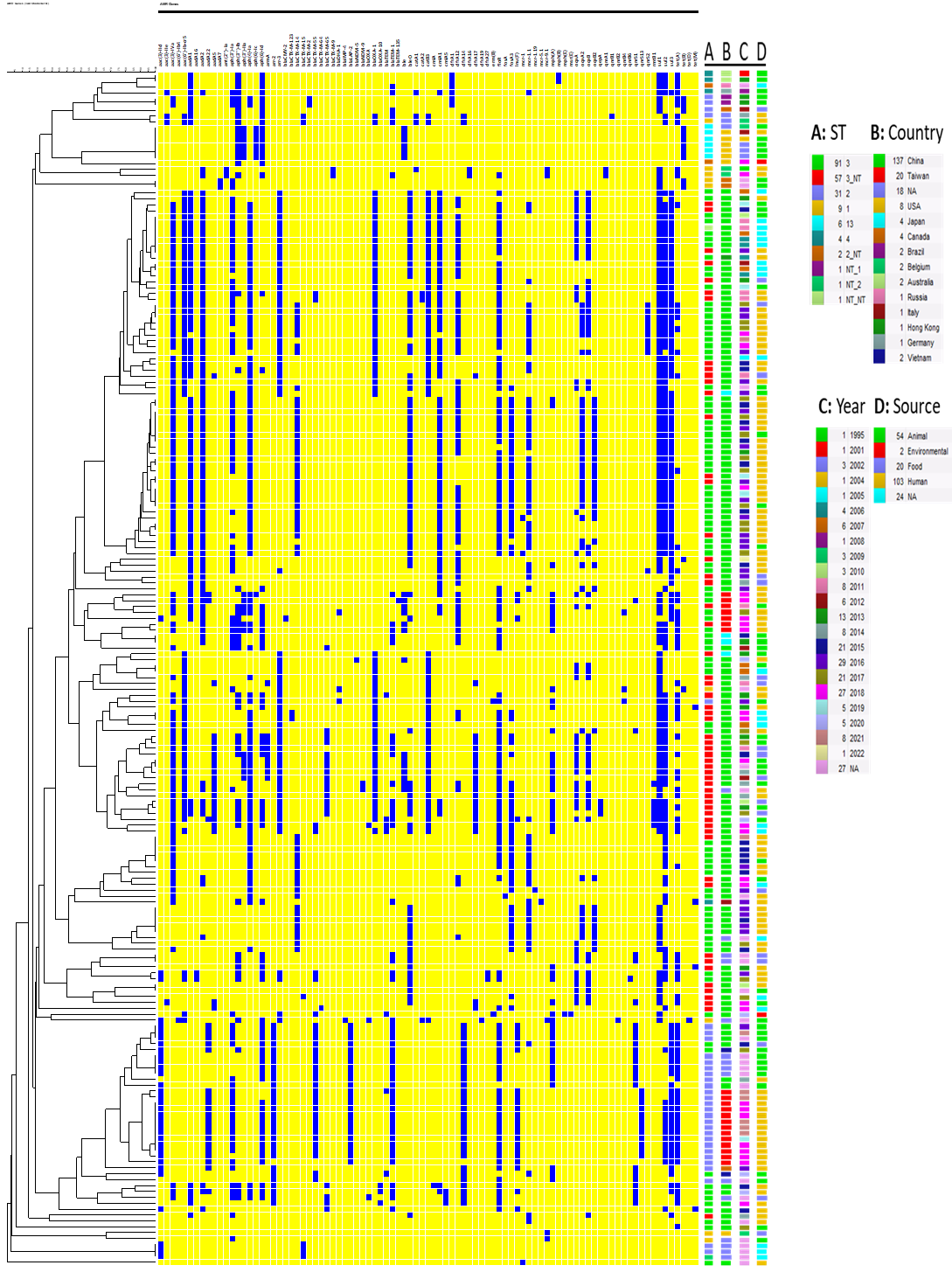


**Figure S4.** Dendrogram of plasmids originating from *Salmonella* strains based on the AMR gene profiles. Clustering was performed using Anderberg correlation of numerical values to generate the dendrogram using the unweighted pair group means with averages (UPGMA) algorithm. The 91 AMR genes are listed in alphabetical order from left to right and detailed data for blue (AMR gene present) and yellow (AMR gene absent) are shown in Table S2. The columns to the right blue/yellow AMR data display the demographical data for the plasmids, the legend for the colors of the boxes is displayed in the figure.

**
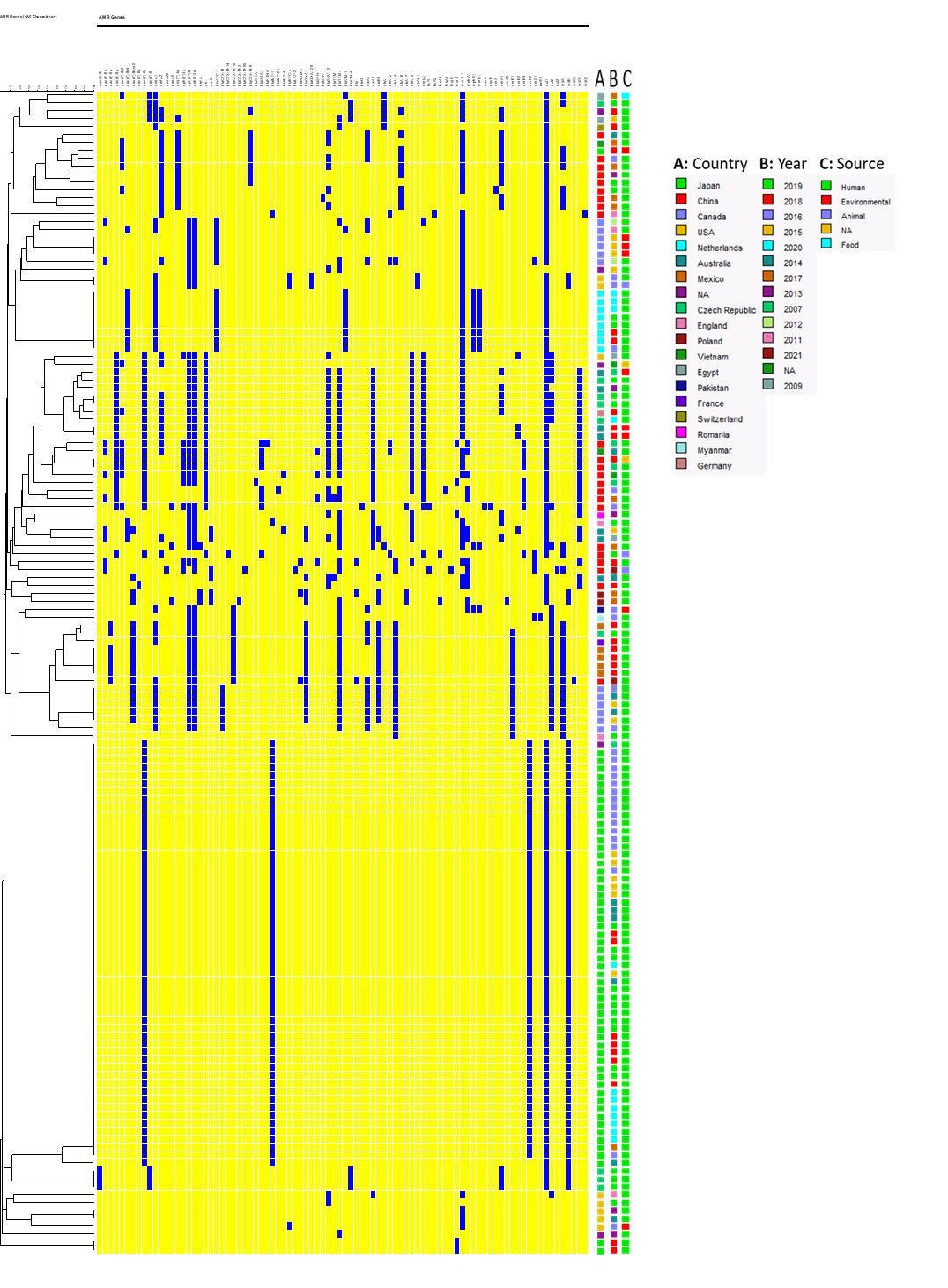
**

**Figure S5.** Dendrogram of plasmids originating from *Enterobacter hormaechei* isolates in the study strains based on the AMR gene profiles. Clustering was performed using Anderberg correlation of numerical values to generate the dendrogram using the UPGMA algorithm. The 88 AMR genes are listed in alphabetical order from left to right and detailed data for blue (AMR gene present) and yellow (AMR gene absent) are shown in Table S2. The columns to the right blue/yellow AMR data display the demographical data for the plasmids, the legend for the colors of the boxes is displayed in the figure.

**
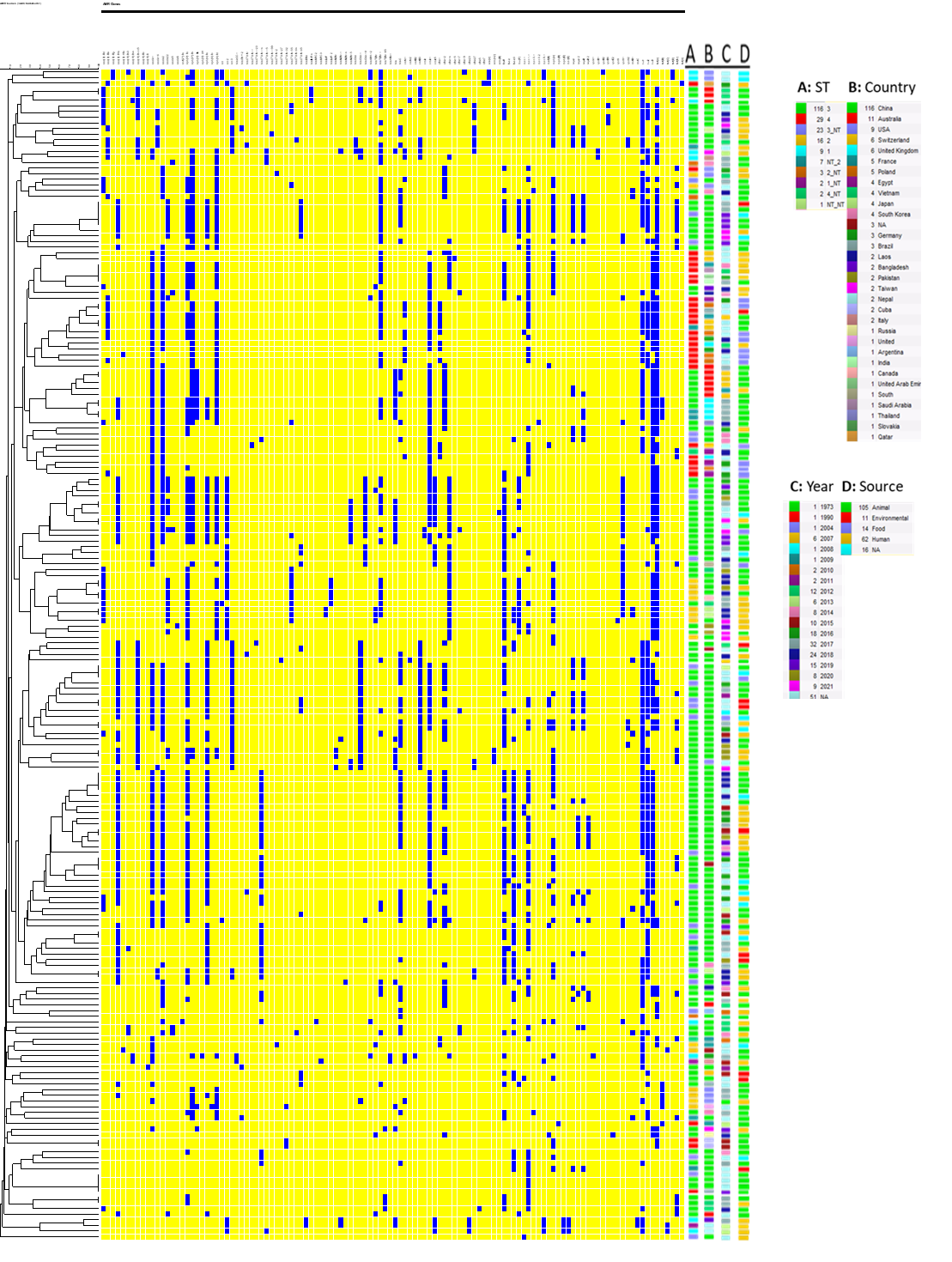
**

**Figure S6.** Dendrogram of plasmids originating from *Escherichia* strains based on the AMR gene profiles. Clustering was performed using Anderberg correlation of numerical values to generate the dendrogram using the UPGMA algorithm. The 118 AMR genes are listed in alphabetical order from left to right and detailed data for blue (AMR gene present) and yellow (AMR gene absent) are shown in Table S2. The columns to the right blue/yellow AMR data display the demographical data for the plasmids, the legend for the colors of the boxes is displayed in the figure.
